# Inadequacy of gestational weight gain during high-risk pregnancies is not associated with household food insecurity

**DOI:** 10.1186/s12884-021-03950-y

**Published:** 2021-06-29

**Authors:** Aléxia Vieira de Abreu Rodrigues, Ana Lúcia Pires Augusto, Rosana Salles-Costa

**Affiliations:** 1 Institute of Nutrition Josué de Castro, Federal University of Rio de Janeiro, Av Carlos Chagas Filho, 373, CCS. Bloco J, 2° andar. 21941-902 - Ilha do Fundão, Rio de Janeiro, Brazil; 2 Nutrition Faculty Emília de Jesus Ferreiro, Federal Fluminense University, Rua Mário Santos Braga, 30, 4° andar, 24020-140 - Niterói, Rio de Janeiro, Brazil

**Keywords:** Gestational weight gain, Pregnant women, Food and nutrition security, Social determinants of health

## Abstract

**Background:**

Inadequate gestational weight gain (GWG) is associated with adverse outcomes in maternal and child health and can be enhanced by social inequalities, such as lower education and household food insecurity (HFI). Women are more vulnerable to HFI, which has been associated with negative health effects for pregnant women during the prenatal and puerperal periods, particularly in regard to the aggravation of pregnancy risks. This study investigated the association between sociodemographic characteristics and HFI with respect to adequacy of total GWG among women with high-risk pregnancies.

**Methods:**

This was a prospective cohort study that evaluated the total GWG of 169 pregnant women. The women were seen at a public university hospital in the metropolitan region of Rio de Janeiro (Brazil). Their sociodemographic and gestational characteristics and the Brazilian Scale of Domestic Food Insecurity were investigated. To estimate the total GWG, the difference between the patient weight at the last prenatal visit and the initial patient weight was verified, with both collected from the medical records of the pregnant women. The classification of the total GWG considered the recommendations of the Institute of Medicine (IOM) (2009). A multinomial logistic regression model assessed the risk (odds ratio; OR) and confidence intervals (CI 95%)) of insufficient and excessive GWG with exposure to HFI and other covariates (*p* value <0.05).

**Results:**

Insufficient and excessive GWG were observed in 27.8% and 47.9% of the pregnant women, respectively. More than half of the women (74.6%) had a high education level. Exposure to mild HFI occurred in 44.2% of the women. After adjustment, the HFI was not associated with insufficient or excessive GWG. The educational level of women was the only variable significantly associated with a lower risk of GWG insufficiency (OR: 0.10; 95% CI: 0.01–0.89).

**Conclusions:**

In this population, higher maternal education was a protective factor against insufficient GWG. We highlight the importance of additional health support and counseling for women in the most vulnerable social conditions, considering the importance of access to information for reducing health risks.

## Background

Weight gain during pregnancy is one of the main markers of physiological and somatic changes that reflect fetal development and growth [1, 2]. However, insufficient or excessive gestational weight gain (GWG) can threaten maternal and child health [3, 4], contributing to the presence of unfavorable gestational outcomes, particularly in women classified as having high-risk pregnancies [5].

As determinants of maternal health, sociodemographic conditions, such as limited access to health care [6–8], also play an important role during pregnancy [9]. Unfavorable aspects, such as less education, the absence of paid work and a low family income, are described in the literature as social determinants associated with insufficient or excessive GWG [2, 9]. Additionally, the evidence supports the hypothesis that household food insecurity (HFI) can be a particularly important barrier to maintaining health during pregnancy [10]. Studies performed to assess the effects of HFI on weight gain during pregnancy observed that HFI was related to both the possibility of insufficient weight gain [11] and metabolic adaptations that increase the risk of obesity [12].

HFI is defined as the lack of secure access to sufficient amounts of safe and nutritious food for normal growth and development and an active and healthy life [13]. Psychometric scales contribute to the assessment of HFI, which can be measured based on concerns about a lack of food to the restriction and experience of hunger in cases of more severe limitations [14, 15].

It is important to note that women are more vulnerable to HFI. The global assessment of the impact of HFI showed that women were approximately 27% more likely to be exposed to more severe HFI than men [16]. In Brazil, data from the last Family Budget Survey (in Portuguese- *Pesquisa de Orçamentos Familiares*) revealed a vulnerability and limited access to food in households headed by women, approximately 52% of whom were affected by severe HFI [17]. A similar finding was reported in the study by Lignani et al. (2020), which evaluated a model of HFI determinants. According to the authors, when the head of the family was female, there was a greater probability of having occupations with lower income levels [18]. The relationship between women and HFI is the result of unequal access to and control of financial resources and unstable incomes affecting the acquisition of food [19].

Additionally, the main causes of HFI for women have been revealed by different sources, such as poverty, low education, being out of the labor market, a large family, the number of children under five, low food diversity and a low frequency of daily meals [16]. During pregnancy, HFI can contribute to nutritional problems related to deficiencies in food intake during a stage of a woman’s life with a higher demand for nutrients for fetal growth and development. HFI is a potential factor associated with the health risks of pregnant women and can contribute to the aggravation of disorders associated with gestational risk, such as anemia and gestational diabetes mellitus [10, 12]. A systematic literature review showed that the associations between HFI and the health of pregnant women were related to poor dietary quality, dietary diversity and inadequate nutrient intake; moreover, HFI was related to higher risks of depression and anxiety [20].

Although HFI is an important measure to be evaluated during pregnancy, few studies have explored its effects on the adequacy of GWG in at-risk women during pregnancy. The investigation of this relationship may assist in the identification of pregnant women exposed to unfavorable social health conditions and development of interventions to reduce the pregnancy risk related to insufficient GWG and excessive GWG. This study aimed to verify the association of HFI and unfavorable sociodemographic characteristics with total GWG inadequacy in high-risk pregnant women.

## Methods

### Study design

This prospective cohort study was carried out with pregnant women from a maternity and prenatal unit of a public university hospital (UH) located in the metropolitan region of Rio de Janeiro (Brazil).

The UH has a coverage area comprising seven municipalities in the metropolitan region of the state of Rio de Janeiro [21]. Comparing the characteristics of these municipalities, it is possible to perceive different sociodemographic conditions. In 2013, the evaluation of the municipal human development index (in Portuguese*: Índice de Desenvolvimento Humano Municipal - IDHM*), for example, indicated that in the municipality where the UH is located, the IDHM was 0.837, the highest index among the seven municipalities. On the other hand, among the municipalities served by the UH, the lowest IDHM rate was 0.654. The IDHM is a number from 0 to 1, where 1 represents greater human development in the locality, and the measure is composed of three dimensions: longevity, income and education. In the municipality where the UH is located, life expectancy at birth was 76.2 years; the average per capita monthly income in real (R$) was R$2.000 (United States dollar-USD 925.9); and among the population over 25, 2.6% were illiterate, and 33.7% had the highest level of education. In contrast, the municipality with the lowest IDHM had a life expectancy at birth of 72.6 years, with an average per capita income of R$ 440 (US$ 203.7); in addition, 11.1% of the population was illiterate, and only 3.7% had the highest level of education [22]. This information indicates the differences and inequalities among pregnant women attending UH.

In Brazil, all pregnant women are entitled to access prenatal care in the Unified Health System by the Primary Health Care Service (PHC). At the PHC, pregnant women are monitored periodically, and when any pregnancy risk is identified and diagnosed, they are referred by the National Regulation System (in Portuguese: National Regulation System - SISREG)^1^ to a more complex prenatal service. The classification of gestational risk is a dynamic process that identifies pregnant women who need interventions that require greater assistance and technological resources and more complex care according to their risk potential, health problems or degree of suffering [6]. The gestational risk factors considered for referral to high-risk prenatal care may be related to previous clinical conditions (heart diseases, hypertension, autoimmune diseases, diseases of the endocrine system), factors related to their previous reproductive history (eclampsia, habitual abortion, perinatal death in a previous pregnancy) and factors related to the current pregnancy (twins, specific hypertensive gestational syndrome, fetal growth retardation) [6].

**Fig. 1:**
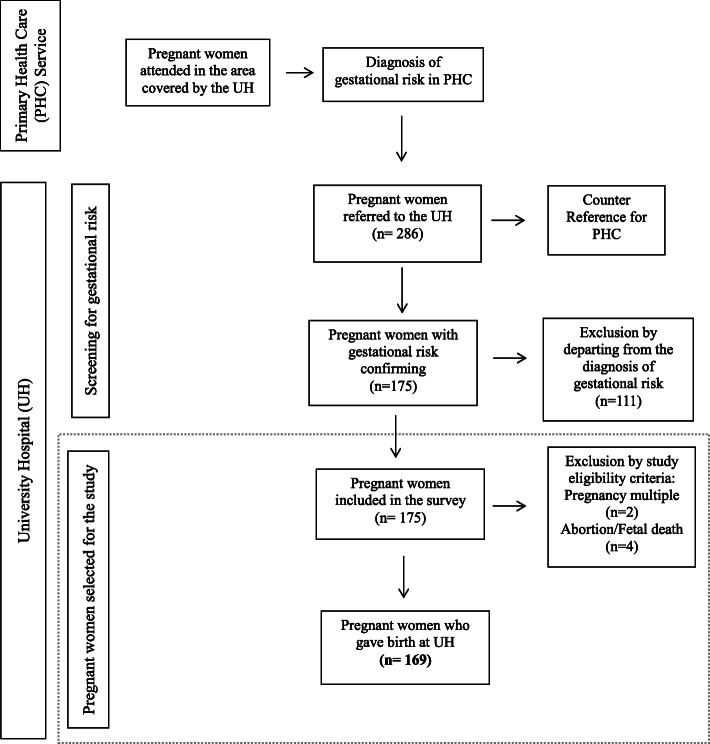
Flowchart for selecting the sample of pregnant women at risk in a university hospital (UH). Metropolitan area of Rio de Janeiro, Brazil, 2019

The UH prenatal and maternity service specifically serves pregnant women referred and classified as having some gestational risk. Pregnant women are referred to a screening consultation to confirm the diagnosis of pregnancy risk. Nonconfirmation indicates the counter-reference, that is, the return of these pregnant women to the primary PHC, whereas confirmation of gestational risk indicates continuity of care in the most complex prenatal care units. In this study, prenatal and maternity ambulatory care was performed at the UH. All of the referred pregnant women who had a proven gestational risk and remained in the UH were approached, and those who consented to participate in our study were included in the sample. Figure 1 shows the flow of care of these pregnant women from the identification of gestational risk in PHC to the possible outcomes in prenatal care regarding the diagnosis of gestational risk until delivery at the UH maternity ward.

The data collection for this study was carried out between August 2017 and October 2019 for the sample selection in addition to the pregnant women presenting with some confirmed gestational risk. The eligibility criteria were age ≥18 years, a single pregnancy and an expected delivery through October 2019.

This study complies with the Declaration of Helsinki [23]. This study was approved by the Research Ethics Committee of the Federal University of Rio de Janeiro (process number: 63737316.5.0000.5257).

### Variables included in this study

#### Gestational weight gain

The total GWG was calculated as the difference between the final weight and the initial weight. The GWG is a measure for assessing and monitoring the health of pregnant women carried out by health professionals in several prenatal units, hospitals and maternity units [6]. The recommendation of the total GWG of the Institute of Medicine (2009) was adopted, so it was necessary to calculate the initial body mass index (BMI) by the weight and height ratio [6, 24].

Height and weight measurements at the beginning of pregnancy were collected from the pregnant woman's health booklet, a document used in prenatal care for monitoring and providing health guidelines during pregnancy. These were measured during the prenatal consultation at the PHC of origin of the pregnant women. Most of the pregnant women included in this sample (77.3%) had these measures reported in the first gestational trimester (1-13 gestational weeks), an opportune time to check their initial weight [6].

The first prenatal consultation of the UH for approximately 76% of the pregnant women occurred after the first gestational trimester, a characteristic that was expected, since some of the risk factors appear or are diagnosed only after the first gestational trimester [6]. In this study, we chose to use the weight measured in the first trimester of pregnancy and recorded in the pregnant woman's health booklet as the initial weight. Thus, even if the consultation of the pregnant woman at UH took place after the first trimester of pregnancy, we considered the initial weight measurements measured at PHC until the 13th week of pregnancy. *initial BMI*

During the first prenatal consultation at UH, an interview was carried out to apply the data collection questionnaire. At this moment, the pregnant woman's health booklet was requested to verify the initial anthropometric measurements of height and weight, and they were considered to estimate the initial BMI (weight [kg]/height m^2^) [6]. For pregnant women who started prenatal care at the PHC after the first gestational trimester and without data about the initial weight registered in the pregnant woman's health booklet (22.7%), self-reported initial anthropometric measurements were requested from them and considered in the analyses. This assessment considers the premise that the measurement of the initial weight represents the weight measured in a period of up to two months before pregnancy and during pregnancy at the limit of 13 weeks of gestation [6]. To determine the final weight, the medical records of the pregnant woman in the maternity ward were consulted. The final weight was considered to be the weight measurement at the last prenatal consultation at UH, two weeks before the date of delivery. In the absence of information about this measure in the medical record, the women were asked about it in the maternity ward after delivery, and the self-reported final weight was considered (8.3%).

The initial BMI was categorized as follows: (i) low weight (BMI <18.5 kg/m^2^); (ii) normal weight (BMI 18.5-24.9 kg/m^2^); (iii) overweight (BMI 25.00-29.9 kg/m^2^); and (iv) obese (BMI ≥30.0 kg/m^2^). The total GWG was classified into three categories of weight gain (adequate, insufficient and excessive) according to the ranges established for the initial BMI: (i) low weight – 12.5-18.0 kg of total GWG; (ii) normal – 11.0-16.00 kg of total GWG; (iii) overweight – 7.0-11.5 kg of total GWG; and (iv) obese – 5.0-9.0 kg of total GWG [17]. The total GWG was adjusted according to the gestational age as a measure of the final weight.

*Household Food insecurity*

HFI was estimated based on the Brazilian Household Food Insecurity Measurement Scale (in Portuguese: *Escala Brasileira de Insegurança Alimentar-EBIA*). The EBIA is a validated instrument for the Brazilian population that investigates the dimensions of access to food through the perception of a family member regarding the lack of financial resources available for the purchase of food [14]. This psychometric scale comprises 14 items and categorizes households according to HFI exposure, ranging from concern about the lack of food at home and insufficient quality of meals due to a lack of food and hunger. The HFI severity levels are measured in the EBIA by the sum of affirmative responses to each item on the scale (8 items for households with adults only and 14 items for households with at least one person less than 18 years old). The absence of positive responses characterizes the condition of household food security (HFS), which indicates that the respondent has no concern or perception of income limitations regarding food purchase and access. The cutoff points classify the HFI into three levels: (i) mild HFI; (ii) moderate HFI; and (iii) severe HFI. More details about EBIA have been described in the literature [14, 25–27]. In this study, the levels of moderate HFI + severe HFI (moderate/severe HFI) were considered in the same category.

*Sociodemographic and health for pregnancy variables*

Sociodemographic and gestational characteristics were included to describe the sample and to investigate social vulnerabilities that could contribute to health inequalities and negatively affect GWG. Maternal age in years was investigated and categorized (<35 years; ≥35 years), considering that pregnant women aged > 35 years tend to have more unfavorable gestational outcomes [6]. The level of education was classified into three categories: <9 years (did not complete elementary school); 9-15 years (completed elementary school or completed high school); and > 15 years (completed high school). Despite the lowest level of education (<5 years of regular study) being a risk factor for pregnant women [6], the categories established in this study considered the highest level of education of the sample of pregnant women, and we classified them by using a category of less education as incomplete primary education (<9 years old). Schooling was described in association with health conditions and GWG [28, 29]. Unsafe marital relationships also compromise the health of pregnant women [6]. The variable marital status considers the impact of living alone and the difficulties faced by single women and mothers in accessing material and financial resources due to gender inequality [17]. In this evaluation, the categories were established (single/separated/widowed; married/stable union) [28]. Drinking water consumption (yes; no) was studied as a gestational risk factor reflecting the unfavorable environmental conditions to which pregnant women are exposed [6].

Data on the race/ethnicity variable were self-declared and evaluated in the categories of white or black/mixed race, considering the vulnerability that racial inequality confers on the health of populations [30, 31]. Family income was investigated in relation to the minimum wage, and two categories were studied (≤ 2 minimum wages; > 2 minimum wages). The minimum wage is the minimum amount regulated by law for paid work [32]. Taking 2018 as a reference, the annual value for the minimum wage in Brazil in the current currency (Real; R$) was R$954, which corresponds in this period to USD 261.4 [33, 34] (the exchange rate at the time was USD 3.65 per R$ 1.00). Family income is an important measure to investigate access to resources such as the purchase of food by families [35].

In relation to health, the following information was investigated as determinants of GWG: The number of prenatal consultations (<8; ≥8) was considered in categories established based on the recommendation of 8 or more consultations for the monitoring and health care of pregnant women and babies, including the identification of risk factors and monitoring of GWG [6]. The number of previous pregnancies (1; 2 to 3) was obtained, as studies indicate the highest risk of excessive GWG in multiparous pregnant women [28, 30, 36]. Gestational age at the time of delivery (<40 weeks; ≥ 40 weeks) was also obtained. Initial BMI [24] was assessed in three categories (low weight; normal; overweight/obese); the decision to sum the categories of overweight BMI and obese BMI was due to the sample size. The consumption of alcoholic beverages (yes; no) and smoking during pregnancy (yes; no) were also investigated in relation to GWG [6, 36].

With the exception of the variable total number of prenatal consultations and gestational age at delivery, which were investigated at the maternity hospital, all of the other data were obtained at the first consultation of the pregnant woman at the UH.

### Statistical analysis

We estimated the proportions and confidence intervals (95% CIs) to describe the study variables. In the first step, the chi-square test was used to compare sociodemographic, HFI and pregnancy characteristics between GWG categories (adequate GWG, insufficient GWG and excessive GWG). The variables with a level of significance in this first analysis, defined as *p* < 0.20, were included in the multivariate analysis. The decision to adopt a more conservative level of statistical significance followed the recommendations used in the literature [37, 38]. In the second step, a multinomial regression model was used to verify the variables associated with GWG. We assigned the adequacy GWG category as a reference in relation to the inadequate (insufficient and excessive) GWG categories. The data are expressed as odds ratios (ORs) and 95% CIs. Variables that in the bivariate analysis were associated with GWG, with a significance level of up to 20% (*p* value <0.20), were considered in the adjusted model. In the final model, the level of significance was defined as a *p* value <0.05. All data processing and analysis were performed using the Stata statistical package (version 16) [39].

## Results

During the reference period of this study, 286 pregnant women were referred to their first consultation in the obstetrics sector of the UH prenatal clinic, and 117 (41%) of them were ineligible (Fig. 1). The final sample included 169 women with an average age of 30.2 years, and their race/ethnicity was predominantly black/mixed-race. Most were married or lived in a stable relationship, had completed high school, and had a family income ≤ twice the minimum wage. Regarding the gestational profile, most women attended at least eight prenatal consultations and did not consume alcohol or smoke during pregnancy. More than half of the women included in this study were exposed to some level of HFI. Considering their initial BMI, there was a high proportion of pregnant women with obesity. Inadequate total GWG (insufficient and excessive) was found in most women; 47% of women had excessive GWG, and approximately 28% of pregnant women had insufficient GWG (Table 1).

**Table 1:** Proportion (%) and 95% confidence interval (CI) in pregnant women. University Hospital from a Metropolitan area of Rio de Janeiro, Brazil, 2019

Variables	Proportion (%)	95% CI
Total GWG^a^		
Insufficient	23.7	17.8-31.1
Adequacy	21.9	16.1-29.0
Excessive	54.4	46.5-62.0
Maternal age		
≤ 35 years old	72.8	65.1-79.4
> 35 years old	27.2	20.6-34.8
Race/ethnicity		
White	32.5	25.8-40.0
Black/mixed-race	67.5	60.0-74.1
Marital status		
Single/Divorced/Widowed	28.4	22.0-35.7
Married	71.6	64.3-78.0
Educational level		
< 9 years	16.6	11.6-23.0
9 – 15 years	74.5	67.4-80.6
> 15 years	8.9	5.4-14.2
Consumption of potable water		
Yes	79.0	72.1-84.6
No	21.0	15.4-27.8
Family income		
≤ 2 minimum wages	69.1	61.5-75.8
>2 minimum wages	30.9	24.2-38.4
Level of Security/Food insecurity (FI)		
Food security	43.5	36.1-51.3
Mild FI	44.2	36.7-52.0
Moderate/severe FI	12.3	8.02-18.3
Gestational trimester in first antenatal care PHC^c^		
1^st^ trimester	77.3	69.3-83.7
2^nd^ - 3^rd^ trimester	22.7	16.3-30.7
Gestational trimester in first antenatal care UH^d^		
1^st^ trimester	23.2	17.3-30.3
2^nd^-3^rd ^trimester	76.8	69.7-82.7
Number visits prenatal		
< 8	50.9	43.3-58.4
≥ 8	49.1	41.6-56.6
Number previous pregnancy		
1	34.3	26.7-42.8
2 – 3	46.3	37.9-54.8
≥ 4	19.4	13.5-27.0
Consumption of alcohol		
Yes	7.1	4.1-12.2
No	92.9	87.8-96.0
Current smoker		
Yes	6.5	3.6-11.4
No	93.5	88.6-96.4
Pre pregnancy BMI^b^		
Low weight	2.5	0.9-6.7
Normal	35.3	28.1-43.1
Overweight/ Obesity	62.2	54.2-69.5
Weeks’ gestation at delivery		
< 40 weeks	27.5	20.2-36.2
≥ 40 weeks	72.5	63.7-79.8

^a^Gestational weight gain estimated according Institute of Medicine (2009)

^b^Body Mass Index (Kg/m^2^)

^c^Primary Health Care Service

^d^University Hospital

The categories of gestational weight gain were correlated (*p* value <0.20) with the following variables: maternal age, marital status, educational level, smoking and alcohol consumption during pregnancy, number of prenatal visits, weeks’ gestation at delivery and household FI (Table 2). Insufficient GWG was more common in women with a lower educational level (48.1%), married marital status (34.1%), age up to 35 years (26.6%) and those who reported alcohol beverages consumption (45.4%) and cigarettes (60%). In turn, excessive GWG was more common in women over 35 years old (59.0%), with a single marital status (56.9%) and with a higher educational level (66.7%). Additionally, more common were the number of prenatal consultations ≤ 8 (58.7%) and a gestational age at delivery greater than 40 weeks’ gestation (53.5%) for women with excessive GWG. Regarding FI, exposure to moderate/severe FI was more common for women with both insufficient GWG (31.6%) and excessive GWG (68.4%).

**Table 2:** Association between variables and adequacy and inadequacy (insufficient and excessive) gestational weight gain in pregnancy women. University Hospital from a Metropolitan area of Rio de Janeiro, Brazil, 2019

	Gestational weight gain			
	Adequacy	Inadequacy		*p* value^*^
Variables		Insufficient	Excessive	
	%^a^	%^a^	%^a^	
Maternal age				0.197^**^
≤ 35 years old	21.1	26.6	52.3	
> 35 years old	28.2	12.8	59.0	
Race/ethnicity				0.812
White	19.2	23.1	57.7	
Black/mixed	23.1	24.1	52.8	
Marital status				0.165^**^
Single/Divorced/Widowed	23.3	19.8	56.9	
Married	18.2	34.1	47.8	
Educational level				0.021^**^
< 9	11.1	48.1	40.7	
9-15	24.6	19.5	55.9	
≥ 16	20.0	13.3	66.7	
Consumption of potable water				0.479
Yes	20.0	24.0	56.0	
No	29.4	23.5	47.1	
Family income				0.950
≤ 2 minimum wages	22.7	23.6	53.6	
> 2 minimum wages	20.4	25.0	54.5	
Level of Security/Food insecurity (FI)				0.099^**^
Food security	21.7	23.2	55.1	
Mild FI	30.3	21.2	48.5	
Moderate/severe FI	-	31.6	68.4	
Gestational trimester in first antenatal care PHC^c^				0.928
1^st^ trimester	21.8	20.8	57.4	
2^nd^ - 3^rd^ trimester	20.7	24.1	55.2	
Gestational trimester in first antenatal care UH^d^				
1^st^ trimester	22.2	22.2	55.6	0.971
2^nd^-3^rd^trimester	21.7	24.2	54.2	
Number visits prenatal				0.178^**^
< 8	23.7	17.5	58.7	
≥ 8	20.0	30.0	50.0	
Number previous pregnancy				0.633
1	17.4	28.3	54.4	
2 - 3	27.1	25.4	47.5	
≥ 4	13.0	30.4	56.5	
Consumption of alcohol				0.129^**^
Yes	27.3	45.4	27.3	
No	21.5	22.1	56.4	
Current smoker				0.021^**^
Yes	10.0	60.0	30.0	
No	22.7	21.3	56.0	
Pre pregnancy BMI^b^				0.874
Low weight	25.0	25.0	50.0	
Normal	27.3	21.8	50.9	
Overweight/Obese	19.6	24.7	55.7	
Weeks’ gestation at delivery				0.193^**^
< 40 weeks	33.3	18.2	48.5	
≥ 40 weeks	18.6	27.9	53.5	

^a^Proportions

^b^Body Mass Index (Kg/m^2^)

^c^ Primary Health Care Service

^d^ University Hospital

^*^ Chi squared test

^**^p value <0.20

Table 3 presents the unadjusted and adjusted multinomial logistic regression. In the bivariate analysis, more years of schooling and the woman's age were considered protective factors for insufficient GWG. Living without a partner, being a current smoker and having <8 total prenatal consultations were considered factors that increased the OR of insufficient GWG. An increased risk for insufficient GWG and excessive GWG was observed with an increase in the number of gestational weeks at delivery (> 40 weeks’ gestation). HFI was not related to GWG. After adjusting the model, only the educational level of the pregnant woman was significantly and inversely associated with insufficient GWG; thus, the higher the education level of the pregnant woman was, the lower the risk of inadequate GWG was (*p* value <0.05).

**Table 3:** Odds ratio (OR) and 95% confidence interval (CI) of the association between variables and insufficient and excessive gestational weight gain (GWG) in pregnant women. University Hospital from a Metropolitan area of Rio de Janeiro, Brazil, 2019

	Bivariate model						Adjusted model					
	Insufficient GWG			Excessive GWG			Insufficient GWG			Excessive GWG		
	OR	CI 95%	***p*** value	OR	CI 95%	***p*** value	OR	CI 95%	***p*** value	OR	CI 95%	***p*** value
Maternal age												
≤ 35 years old	Ref.[1]			Ref.[1]			Ref.[1]			Ref.[1]		
> 35 years old	0.36	0.11-1.18	0.09^*^	0.84	0.35-2.00	0.70	0.66	0.13-3.28	0.62	1.37	0.43-4.42	0.60
Marital status												
Single/Divorced/Widowed	2.20	0.8-6.12	0.13^*^	1.07	0.42-2.72	0.88	1.83	0.50-6.68	0.36	0.84	0.28-2.58	0.76
Married	Ref.[1]	Ref.[1]		Ref.[1]	Ref.[1]		Ref.[1]	Ref.[1]		Ref.[1]		
Educational level												
< 9	Ref.[1]	Ref.[1]		Ref.[1]	Ref.[1]		Ref.[1]	Ref.[1]		Ref.[1]		
9-15	0.18	0.05-0.72	0.02^**^	0.62	0.16-2.40	0.49	0.10	0.01-0.89	0.04^**^	0.20	0.02-1.70	0.14
≥ 16	0.15	0.02-1.37	0.09^*^	0.91	0.15-5.58	0.92	0.05	0.002-1.22	0.06	0.18	0.01-2.35	0.19
Level of Security/Food insecurity (FI)												
Food security	Ref.[1]	Ref.[1]		Ref.[1]								
Mild FI	0.65	0.24-1.75	0.40	0.63	0.28-1.43	0.27						
Moderate/severe FI	0.29	0.26-0.32	0.98	0.26	0.23-0.29	0.98						
Number visits prenatal												
< 8	2.03	0.80-5.20	0.14^*^	1.01	0.46-2.22	0.98	4.03	0.94-17.25	0.06	0.95	0.33-2.68	0.92
≥ 8	Ref.[1]	Ref.[1]		Ref.[1]	Ref.[1]		Ref.[1]	Ref.[1]		Ref.[1]		
Consumption of alcohol												
Yes	1.62	0.36-7.33	0.53	0.38	0.07-2.00	0.25						
No	Ref.[1]	Ref.[1]		Ref.[1]								
Current smoker												
Yes	6.37	7.27-55.90	0.09^*^	1.21	0.12-12.09	0.87	5.01	0.36-69.24	0.23	0.92	0.07-11.99	0.95
No	Ref.[1]	Ref.[1]		Ref.[1]	Ref.[1]		Ref.[1]	Ref.[1]		Ref.[1]		
Weeks’ gestation at delivery												
< 40 weeks	Ref.[1]	Ref.[1]		Ref.[1]	Ref.[1]		Ref.[1]	Ref.[1]		Ref.[1]		
≥ 40 weeks	2.75	0.85-8.94	0.09^*^	1.98	0.76-5.14	0.16^*^	2.13	0.49-9.29	0.31	2.10	0.69-6.43	0.19

^ [1]^Reference category

^*^p value <0.20

^**^p value <0.05

## Discussion

Our study showed that inadequate GWG (insufficient or excessive) was not associated with exposure to FI during pregnancy in this sample of women with high-risk pregnancies. However, after assessing the sociodemographic and health conditions of pregnant women, a higher level of education was a protective factor against insufficient GWG.

This study demonstrated a higher prevalence of women with excessive GWG. An analysis of secondary data from multicenter studies in the United States involving 8293 women showed that 73% had excessive GWG, corresponding to approximately three in four women [40]. Zhao et al. [4] identified approximately 50% of pregnant women with excessive GWG in a study conducted in China, and 15.2% had insufficient GWG. In Brazil, Campos et al. [41] evaluated the adequacy of GWG among pregnant women in the northern region of the country and observed a prevalence of excessive GWG in almost half of the sample (59%) and insufficient GWG in 19% of women. A similar result was observed for pregnant women in Rio de Janeiro; according to the authors, almost 50% of women evaluated had excessive GWG, and less than 30% had adequate GWG [42].

A direct determinant of GWG is the initial BMI [14]. In this study, no associations were observed between these variables. However, we observed that more than half of the pregnant women had an initial BMI corresponding to overweight and obesity. In contrast, a low initial BMI was observed in only 2.6% of the sample, a proportion close to the results of the Brazilian Food and Nutrition Surveillance System (in Portuguese: *Sistema de Vigilância Alimentar e Nutricional* - SISVAN) during 2019, when 2.5% of adult Brazilian women were found to have a low BMI. Data from the Brazilian SISVAN also revealed an increase in the prevalence of overweight BMI among women of reproductive age; 34.2% were considered overweight, and 29.7% were obese based on BMI [43].

In this study, it was also found that more than half of the mothers (56.4%) were exposed to some level of HFI during pregnancy. Oliveira et al. [44] investigated HFI in a group of pregnant women attending PHC in northeastern Brazil and identified a prevalence of 42.7% of HFI in the sample, a value lower than the result in this study. In addition, in the present study, we opted for the sum of the most severe levels of HFI (moderate + severe). Thus, it was observed that 12.3% of women had limited access to food in terms of quantity and quality and possibly continued to experience hunger; this proportion was lower than that reported by Marano et al. [45] when investigating HFI in pregnant women in two cities in Rio de Janeiro (14.9% moderate/severe HFI).

Other studies carried out in Brazil evaluating HFI during pregnancy showed significant variations in the prevalence estimated by the EBIA, depending on the region of the country. In studies carried out in the northeastern and southeastern regions of Brazil, the authors reported a higher prevalence of HFI among pregnant women, ranging from 59% to 71.6% [46, 47] and 37.8% in southeastern Brazil [48]. These differences in prevalence are consistent with the distribution of severe HFI in Brazil. Although a higher prevalence of HFI (56.5%) was observed among the women in this study, no relationship with GWG adequacy was found.

Few studies have evaluated the relationship between GWG and HFI. A meta-analysis identified that pregnant women exposed to home HFI had an increased prevalence of discrepant weight gain, both excessive and inadequate weight gain [49]. Other studies have reported a lack of an association between GWG and HFI, as observed by Laraia et al. [50]. According to these authors, no significant association was found between HFI and GWG among pregnant women in the United States. However, the authors observed that the average weight of women with HFI in the study was higher, as well as the GWG adequacy rate, suggesting HFI as an indicator for excessive GWG [50]. In Brazil, a study of pregnant women in the northeastern region of the country to identify the association among different factors, including HFI status and GWG, showed no significant association with HFI and the GWG outcome; this finding was similar to that of this study [44].

The main finding of this study indicates a reduction in the risk of insufficient GWG for women with more years of schooling, since pregnant women with access to secondary and higher education were more protected from this level of GWG than women with less than nine years of schooling. In the literature, a lower risk of insufficient GWG was also associated with higher levels of schooling [51]. On the other hand, a higher level of education was related to a higher risk of excessive GWG [8, 45].

In addition, higher education has been described as being associated with a greater likelihood of GWG adequacy [28] and a lower likelihood of excessive GWG [52]. Pregnant women with less than four years of education had a risk of excessive GWG that was approximately five times greater than that of other pregnant women [53]. These findings indicate that the increase in years of schooling may act as a protective factor for GWG. In addition, the risk associated with low education and a greater propensity for excessive GWG [54, 55] and insufficient GWG [56] point to the importance of education as a social determinant of health [57]. Thus, understanding that inadequate GWG is a modifiable risk factor and that both excessive and insufficient weight gain during pregnancy can threaten the health of women and their children [58] is important for strengthening health services and factors related to the environment, education and health promotion [59].

This study has some limitations. For the *pregnant woman's health booklet* and height measurements, when these measures were not available, the self-reported data of the pregnant women were considered. In such cases, the possibility of biases attributed to overestimation or underestimation of measures must be considered [60, 61]. Researchers have corroborated the quality of the measures of weight and height measured by anthropometry versus those obtained by self-report in population-based studies. For example, Conde et al. (2013) compared the measured and reported weight and height measurements of the Brazilian population and highlighted that the BMI estimates, measured or reported, were relatively close. In addition, a validation study of self-reported measures found that 84% of women who reported their weight and height measurements were categorized appropriately in regards to their BMI classification [62]. Health behaviors and interventions such as nutritional monitoring, possible interactions of the diet to control gestational weight gain and physical exercise during pregnancy were not evaluated in this study. However, there is some evidence regarding the low impact of these measurements on weight gain during pregnancy [63]. The investigation of family income in terms of minimum wages limited the assessment of income in relation to the number of family members (income per capita).

## Conclusion

In this population, an important factor for GWG is maternal education, among the other determinants evaluated and widely discussed in the literature as predictors of GWG. Additionally, a lower level of education of the pregnant women evaluated may have preceded the effects of HFI on its relationship with the GWG of these women with high risks during pregnancy. Given the risk of inadequate gain during a high-risk pregnancy and given that GWG is a modifiable factor, the importance of additional support and health counseling is highlighted, particularly nutrition education interventions, such as a health-promoting tool for pregnant women and fetuses, particularly for women who are more socially vulnerable and who have low levels of formal education.

## Data Availability

The data sets analyzed during the present study are not publicly available due to the compromised privacy and exposure of the participants, but are available from the corresponding author upon reasonable request.

## Abbreviations

BMI: body mass index
CI: confidence intervals
EBIA: Brazilian Household Food Insecurity Measurement Scale *(in Portuguese: Escala Brasileira de Insegurança Alimentar)*
GWG: gestational weight gain
HFI: household food insecurity
HFS: household food security
IDHM: municipal human development index (in Portuguese: *Índice de Desenvolvimento Humano Municipal*)
IOM: Institute of Medicine
OR: odds ratio
PHC: primary health care
R: Real
SISREG: National Regulation System (in Portuguese: *Sistema Nacional de Regulação*)
SISVAN: Brazilian System of Food and Nutritional Surveillance (in Portuguese: *Sistema de Vigilância Alimentar e Nutricional*)
UH: university hospital
USD: United States dollar

## Footnotes

1 SISREG is a virtual management system of the Ministry of Health that manages from the basic and specialized outpatient network to hospital and hospitalization, at the levels: municipal, state, federal, including private services of the Unified Health System and university students, seeking greater control flow and optimization of the use of resources, in order to guarantee the humanization of services.
